# The Influence of the Addition of Hemp Press Cake Flour on the Properties of Bovine and Ovine Yoghurts

**DOI:** 10.3390/foods12050958

**Published:** 2023-02-24

**Authors:** Gjore Nakov, Biljana Trajkovska, Natalija Atanasova-Pancevska, Davor Daniloski, Nastia Ivanova, Mirela Lučan Čolić, Marko Jukić, Jasmina Lukinac

**Affiliations:** 1 College of Sliven, Technical University of Sofia, 59 Bourgasko Shaussee Blvd., 8800 Sliven, Bulgaria; 2 Faculty of Biotechnical Sciences, University “St. Kliment Ohridski”, 7000 Bitola, North Macedonia; 3 Faculty of Natural Sciences and Mathematics-Skopje, Department of Microbiology and Microbial Biotechnology, Ss. Cyril and Methodius University in Skopje, 1000 Skopje, North Macedonia; 4 Advanced Food Systems Research Unit, Institute for Sustainable Industries and Liveable Cities, College of Health and Biomedicine, Victoria University, Melbourne, VIC 8001, Australia; 5 Teagasc Food Research Centre, Food Chemistry and Technology Department, Moorepark, Fermoy, P61 C996 Cork, Ireland; 6 Faculty of Food Technology Osijek, Josip Juraj Strossmayer University of Osijek, 31000 Osijek, Croatia

**Keywords:** yoghurt, hemp press cake, by-products, bovine milk, ovine milk

## Abstract

Hemp press cake flour (HPCF) is a by-product of hemp oil production rich in proteins, carbohydrates, minerals, vitamins, oleochemicals, and phytochemicals. The purpose of this study was to investigate how the addition of HPCF to bovine and ovine plain yoghurts at concentrations of 0%, 2%, 4%, 6%, 8%, and 10% could change the physicochemical, microbiological, and sensory properties of the yoghurts, focusing on the improvement of quality and antioxidant activity, and the issue of food by-products and their utilisation. The results showed that the addition of HPCF to yoghurts significantly affected their properties, including an increase in pH and decrease in titratable acidity, change in colour to darker, reddish or yellowish hue, and a rise in total polyphenols and antioxidant activity during storage. Yoghurts fortified with 4% and 6% HPCF exhibited the best sensory properties, thus maintaining viable starter counts in the yoghurts during the study period. There were no statistically significant differences between the control yoghurts and the samples with 4% added HPCF in terms of overall sensory score while maintaining viable starter counts during the seven-day storage. These results suggest that the addition of HPCF to yoghurts can improve product quality and create functional products and may have potential in sustainable food waste management.

## 1. Introduction

In the last few decades, the research interest on environment and ecosystem improvements has increased significantly, primarily due to the rise in the world population. The food sector, facing daily challenges, struggles to achieve sustainability, i.e., the valorisation of food by-products. Most often, the purpose of this type of by-product is to be used as animal feed; nevertheless, it is insufficient to create a modern vision of a circular economy [1]. Vegetable oils are important constituents in human nutrition. Sunflower, olive, and rapeseed oils are the most commonly used and commercialised oils (regardless of their possible favourable or unfavourable effect on human health); however, very recently, the hemp oil derived from pressing hemp seeds (*Cannabis sativa* L.), has been promoted as a healthier option, hence its usage trend has been marginally increasing [2]. In addition to the food industry, this plant can also be used in the pharmaceutical industry, particularly the seeds of non-psychotropic varieties of *Cannabis sativa* L. that contain less than 0.3% Δ9-tetrahydrocannabinol (Δ9-THC) [3]. After its processing and oil extraction, the seeds’ by-product contains functional food components rich in proteins, minerals, carbohydrates, vitamins, oleochemicals, and phytochemicals, all of which are found to possess high antioxidant activity [4].

*Cannabis sativa* L. seeds are a valuable source of protein (25–30%), polyunsaturated fatty acids (75–80%), dietary fibre (4%), and phenolic compounds. The hydrolysis of hemp seed proteins by enzymes, such as pepsin, pancreatin, trypsin, and proteases, leads to the release of bioactive peptides with anti-hypertensive, antioxidant, antiproliferative, and anti-inflammatory properties [5].

Hemp press cake flour (HPCF) is a residue obtained from the production of oil from hemp seeds. The process involves cleaning the seeds, grinding them into a paste, and applying high pressure to extract the oil. The resulting solid material, referred to as press cake, is further processed to eliminate remaining oil by drying, grinding, and creating a fine flour. HPCF is a nutritionally rich ingredient containing protein, dietary fibre, minerals, vitamins, oleochemicals, and phytochemicals.

Phytochemicals with high antioxidant activity, particularly flavonoids, have been identified in HPCF after fermentation. These phenolic compounds offer protection against oxidative stress and may lower the chance of chronic diseases. There is a strong link between flavonoid and polyphenol intake and a reduced risk of chronic diseases such as neuro-degenerative diseases, cancer, and cardiovascular disorders [6].

Fermentation enhances the bioactivity and availability of antioxidants in HPCF that remain after oil extraction. Through a series of reactions, fermentation activates bioactive compounds as antioxidants, resulting in cell wall breakdown and the synthesis of various bioactive compounds [7]. Additionally, fermentation boosts the bioavailability of phenolic compounds and simplifies their structure by freeing bound phenols from the matrix and undergoing biotransformation of phenolic compounds [8]. Leonard et al. (2019) [8] have noted that fermenting foods is an effective method for improving their nutritional and functional value. Incorporating HPCF into food products, such as yoghurt, can enhance their nutritional and functional value, increase the bioavailability of bioactive compounds, and provide potential health benefits for consumers [4].

The use of the by-product created upon hemp oil extraction (hemp press cake—HPC) has mostly been aimed at the production of innovative and functional products of plant origin, including gluten-free bread [9], chips [10], sponge cake [11], and vegetable yoghurt [12], to name but a few. The addition of this by-product in food is because it contains large amounts of dietary fibre, hence improving the structure of the fortified food products. On the other hand, a real challenge has been the use of hemp press cake in the production of food products of dairy origin, since its utilisation is still not completely accepted by consumers and the market [13].

Yoghurt is known as a lactic acid, bacteria-fermented dairy product, which is mainly produced from bovine milk. Its consumption by both adults and children makes yoghurt one of the most-used dairy products. Very often, it is also part of the diet of infants due to its protein and essential micronutrients (calcium, potassium, zinc, phosphorus, and magnesium). It is mainly produced from bovine milk; however, ovine and caprine milks have recently also been used for yoghurt manufacturing [14,15,16]. While bovine yoghurt has been extensively investigated, there is scarce information on ovine plain and fortified yoghurts despite their greater nutritional values and health benefits [16]. The addition of different components that can increase the nutritional quality of yoghurt with nutrients that are normally missing in the conventional formulations, including vitamins, fibres, and polyphenols, is a new approach to the production of novel milk fermented functional foods. Previous works presented the effect of the addition of olive oil by-products, cinnamon, mango peel powder, rosehip, coriander, and cumin seeds on the quality of yoghurts [17,18]; however, the importance of HPC in yoghurt production has not been characterised in depth.

In the study by Łopusiewicz et al. (2022b) [17], the use of camelina seed press cake (CPC) as a plant-based alternative to dairy yoghurt was investigated. The CPC was fermented using a yoghurt starter culture, resulting in significant acidification, increase in bioactive compounds and antioxidant activity, as well as improved sensory attributes compared to dairy yoghurt. The fermented CPC-based beverage showed potential as a dairy-based yoghurt substitute, with similar textural and rheological properties, higher viable bacterial count, and a high content of bioactive compounds, such as polyphenols and flavonoids. The study concluded that the CPC-based yoghurt-like beverage has the potential to be a new functional product suitable for vegans and vegetarians. In another study, Łopusiewicz et al. (2022a) [18] explored the use of hemp press cakes (HPC) as a raw material for the production of high-value beverages using yoghurt (YO 122) and kefir (commercial grains) starter cultures. The study determined changes in pH, acidity, lactic acid bacteria (LAB) and yeast population, bioactive compounds, and antioxidant activity. The results showed the feasibility of producing high-value beverages with high fermentation efficiency, high survivability of LAB and yeast, and acidification, as well as stability of hemp protein and changes in polyphenolic content. This study provided new insights into the fermentation mechanisms of hemp cake and the differences between yoghurt-like and kefir-like samples, offering a new way to manage HPC as an oil industry residue for the production of dairy-free beverages.

Since the use of HPC proteins (found in the by-product after oil extraction) as a source of nutritional enrichment in food products has gained increasing attention in recent years, more research is needed to fully understand the chemical composition and health benefits of hemp-enriched products [19]. Previous research has explored the impact of HPCF on the nutritional and antioxidant properties of yoghurt; this study places emphasis on monitoring the quality of the yoghurt during storage to determine the long-term effects of incorporating HPCF. By systematically evaluating the impact of HPCF on the quality, antioxidant activity, and sensory properties of both bovine and ovine yoghurt, this study seeks to provide valuable insights into the potential of using food by-products as a fortifying ingredient to improve the functional properties of yoghurt and advance sustainable food production.

Łopusiewicz et al. (2022a) [18] highlight the addition of HPCF to yoghurt and kefir as a new trend in food technology, focusing on enriching existing products with by-products rich in biologically active substances and improving the nutritional value of new products. The authors emphasize that the use of by-products in the creation of new products aligns with the principles of the circular economy and the idea of zero waste.

The objective of a previous study by Nakov et al. (2023) [20] was to explore the use of secondary product HPCF derived from hemp oil production and polyfloral honey as fortifying ingredients in bovine yoghurt. The aim was to reduce waste from hemp oil production by utilizing HPCF as a valuable raw material to improve the functional properties of probiotic yoghurt, contributing to sustainable food production and the use of food by-products in the food industry. The results showed the potential for HPCF to enhance the functional properties of bovine yoghurt.

The present study builds upon the previous research by expanding the scope to include ovine yoghurt and by conducting a systematic evaluation of the impact of HPCF on the quality, antioxidant activity, and sensory properties of both bovine and ovine yoghurt. Unlike the previous study, which only analysed the quality of bovine yoghurt with HPCF added, this study also includes an analysis of ovine yoghurt, and the quality of both types of yoghurt is monitored throughout storage. The study aims to determine the effectiveness of HPCF as a fortifying ingredient, its potential to improve the functional properties of yoghurt, and the optimal concentration of HPCF that can be added while still preserving the quality, antioxidant activity, and sensory properties of the yoghurt. By addressing these objectives, the study aims to promote sustainability in the food industry by utilizing food by-products and reducing waste.

## 2. Materials and Methods

### 2.1. Reagents

All biochemical and chemical reagents were purchased from Sigma-Aldrich, Schnelldorf, Germany, unless otherwise specified.

### 2.2. Raw Materials and Yoghurt Production

Approximately 20 L of both raw bovine milk (Macedonian Holstein Friesian breed) and raw ovine milk (Macedonian Ovchepolian breed) were gifted by a private dairy industry in Bitola, North Macedonia. Samples were stored at 4 °C and were immediately transported to the laboratory of milk and dairy products at the Faculty of Biotechnical Sciences, Bitola, North Macedonia. By using Lactoscan MCCW-V1 (ultrasonic analyser, Milkotronic Lactoscan, Varna, Bulgaria), the chemical composition of both raw full fat milks was determined (Table 1).

Following this, raw milk samples were subjected to thermal treatment at 85 ± 1 °C (Weck Inc., Luray, VA, USA) for a duration of 30 min, as described by Nguyen et al. (2018) [16]. Upon completion of the pasteurization, the samples were cooled to 43 ± 1 °C. After reaching the targeted temperature, 2% of yoghurt starter cultures (ABT-5, CHR Hansen, Hørsholm, Denmark) were added to the samples, as per the manufacturer’s guidelines (500U/2500L), and thoroughly mixed for 40 s at 1200 rpm (IKA EUROSTAR 20 overhead stirrer, Werke GmbH, Köln, Germany).

The HPCF was produced using hemp cake, which was obtained as a by-product during the production of oil from hemp seeds. The hemp seeds used were of the variety Cannabis sativum var. Finola and were sourced from a local producer (Cannabio d.o.o. Croatia). The methodology for obtaining HPCF was described in detail by Nakov et al. (2023) [20]. The process involved drying the press cake obtained after oil extraction, grinding it into a fine flour, and sifting to obtain the desired fraction. The fraction <200 μm was selected for further analysis. The HPCF contained 11 g/100 g lipids, 3.7 g/100 g carbohydrates, 34 g/100 g fibers, and 40 g/100 g protein.

The experiment involved the addition of varying quantities of HPCF (2 g/100 g, 4 g/100 g, 6 g/100 g, 8 g/100 g, and 10 g/100 g) into sterile containers. The inoculated milk was then divided into these containers, mixed, and incubated (yogurt maker Y 140, Elecrem, UK) at a constant temperature of 43 ± 1 °C. The incubation was continued until the control bovine yoghurt samples, which did not contain HPCF, reached a pH of 4.5 ± 0.1 (230 min), and at that moment, incubation was interrupted for all other samples. During the fermentation process, the pH of the milk was constantly monitored using a pH meter (Testo SE & Co. KGaA, Lenzkirch, Germany).

**Figure 1:**
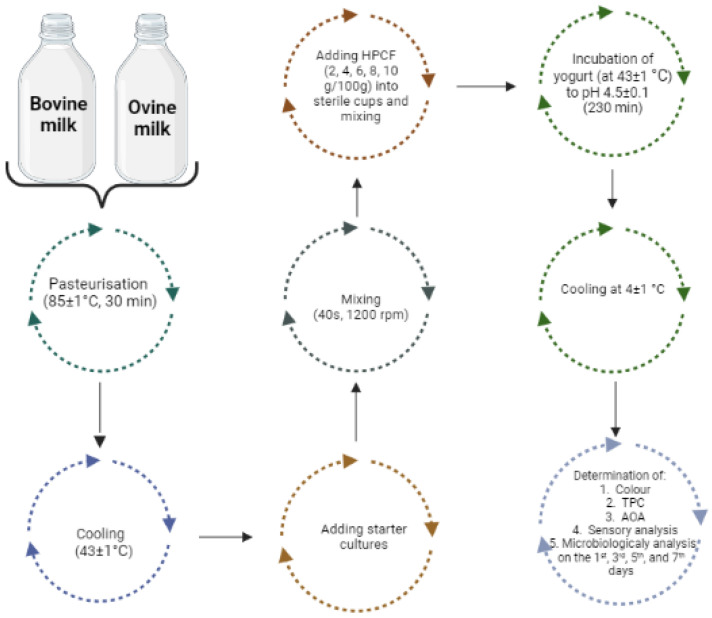
A schematic illustration of the yoghurt production steps (plain and fortified with hemp press cake flour) and the analysis performed on the yoghurt samples.

When the incubation was completed, the yoghurt samples were subjected to cooling (4 ± 1 °C) to prepare them for subsequent analysis. This analysis involved evaluating the samples’ physicochemical, microbiological, and sensory properties (as shown in Figure 1). The evaluations were conducted over a 1-week period, with measurements taken every other day (on the 1st, 3rd, 5th, and 7th days).

All yoghurt samples were made in triplicate batches. The control yoghurt samples without HPCF were referred to as plain samples, while the yoghurt samples with HPCF (2 g/100 g, 4 g/100 g, 6 g/100 g, 8 g/100 g, and 10 g/100 g) were considered fortified samples.

### 2.3. Changes of Titratable Acidity

Titratable acidity was determined by the method described by De Marchi et al. (2009) [21]. Briefly, 10 g of the sample was dissolved in 30 mL of distilled water and mixed thoroughly. A few drops of 2% phenolphthalein indicator (1 mL) were added to the mixed solution. The solution was titrated against standard 0.1 M sodium hydroxide until a pale pink colour persisted for approximately 15 s for complete neutralisation. Titratable acidity was recorded as a Soxhlet–Henkel degree (°*SH*) using a Crison Compact D meter (Crison Instruments SA, Alella, Spain) and is given in the equation below.

(1) °SH=a·2·F

where: *a* = volume (mL) of 0.1 M sodium hydroxide used for titration, *F* = factor of 0.1 M sodium hydroxide.

### 2.4. Colour Determination

The evaluation of the samples’ colour was carried out using a colorimeter (Konica Minolta, Chroma Meter, CR400, Japan). The instrument was standardised using standard white plates. An average value was determined by taking observations from 5 different points (3 on the top and 2 on bottom) of the same sample, and the CIE L* (brightness), a* (+ red/− green) and b* (+ yellow/− blue) parameters were registered according to CIE1976 colour system [22].

### 2.5. Determination of Total Polyphenols and Antioxidant Activity

Measurement of total polyphenolic content was performed following the Folin–Ciocalteu method by measuring absorbance at 760 nm using a UV-VIS Spectrophotometer, (UV-1800, Shimadzu, Japan) [20]. The results were expressed as µg gallic acid equivalent (GAE) per mL of fresh weight (FW) of the sample. The free radical scavenging activity of yoghurt samples was evaluated according to the method described by Barkallah et al. (2017) [23], utilising as an indicator the 1.1-diphenyl-2-picrylhydrazil (DPPH).

### 2.6. Microbiological Analysis

Regarding the microbial counts, 10 g of each yoghurt stored at refrigerator temperature was homogenised in 90 mL of a diluent solution (0.85% sodium chloride and 0.1% tryptone) and serial 10-fold dilutions were prepared. The total mesophilic count excluding lactic acid bacteria was placed onto plate count agar and subsequently incubated at 30 °C for 48 h [24]. *Lactobacillus* spp. was enumerated on de Man–Rogosa–Sharpe agar acidified at pH 5.2 according to ISO 7889 standards [25]. *Streptococcus* spp. was enumerated onto M17 agar supplemented with lactose (5 g/L) and incubated under aerobic conditions at 45 °C for 24 h [25]. The number of *Bifidobacterium* spp., *Enterobacteriaceae*, *Escherichia coli*, *Staphylococcus aureus*, and *Coagulase*-positive were determined using established ISO methods [26,27,28,29]. Yeasts and moulds were enumerated according to the ISO method [30]. Colony-forming units (CFU) were counted on petri dishes expressed as log CFU/mL of yoghurt.

### 2.7. Sensory Evaluation

The sensory evaluation of the yoghurts was performed using a combination of weighted points and hedonic scale analysis [31], as well as a slightly modified method introduced by Nakov et al. (2023) [20]. A panel of 20 semi-trained assessors participated in the sensory evaluation. The yoghurts were equilibrated at room temperature, poured into identical laboratory beakers, and encoded. The yoghurts were given points from 1 to 9 for the attributes including aroma, flavour, texture, aftertaste, and appearance. Each attribute of the yoghurt sample had its own weighting factor thus, the flavour and the aftertaste had the highest weighting factors (1.5 and 1, respectively), while texture, aroma, and appearance had lower weighting factors (0.9, 0.4, and 0.2, respectively). The average grade of each yoghurt was multiplied by the weighting factor representing each attribute and, using this method, the maximum points one yoghurt could reach was 20 [32]. In addition, at the end of the study the 20 evaluators performed a 9-point hedonic scale analysis to evaluate the acceptance of the yoghurts on a scale from 1 indicating “extremely dislike” to 9 meaning “extremely like” [22]. The sensory analysis was performed only on the first day after the yoghurt production. The study was conducted in accordance with Resolution No. R2-0209/2022 of the Ethics Committee of College of Sliven, Technical University of Sofia, which follows the guidelines on ethics and food-related research defined by the European Union, and informed consent was obtained from the participants [33].

### 2.8. Statistical Analysis

Statistical analysis was performed using factorial analysis of variance (3-way ANOVA) followed by Tukey’s honestly significant difference (HSD) test to compare the main effects (yoghurt type, HPCF addition, and storage) and the interaction effects between them on all analysed traits, except for sensory analysis, for which 2-way ANOVA was used. The statistical analysis was performed using XLSTAT software version 2019.2.2 (Addinsoft, New York, NY, USA). The level of significance was set at *p* ≤ 0.05.

## 3. Results and Discussion

### 3.1. Change of pH

**Figure 2:**
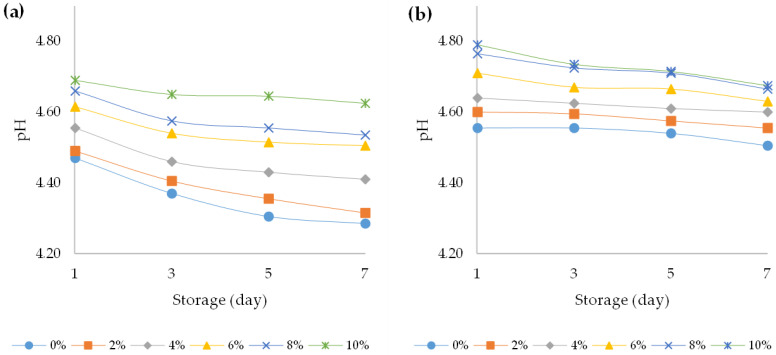
Changes in pH values over the course of storage days for (**a**) bovine and (**b**) ovine plain and fortified yoghurts with different percentages of HPCF.

On the first day, the pH of the plain yoghurts (without HPCF) was 4.47 and 4.56 for bovine and ovine yoghurts, respectively (Figure 2a,b). The addition of HPCF significantly increased the pH value of the yoghurts, regardless of the milk origin (*p* < 0.05); namely, the yoghurts with 10% HPCF had a pH of 4.75 and 4.83 for bovine and ovine yoghurts, respectively, on the first day of storage.

Nevertheless, the pH value of the plain and fortified yoghurts with HPCF decreased with the storage time (*p* < 0.05), thus on the last day of examination for the plain yoghurts it counted 4.29 (bovine plain yoghurt) and 4.51 (ovine plain yoghurt) and for the HPCF fortified yoghurts it was 4.63 (10% HPCF fortified yoghurt) and 4.66 (10% HPCF fortified yoghurt). The decrease in pH value was observed during storage, regardless of the addition of HPCF. A three-way ANOVA revealed that all main effects were statistically significant (*p* < 0.05) (Table S1). The main effect for yoghurt type yielded an F ratio of F (1, 96) = 388.2 (*p* < 0.001), indicating that pH values for ovine yoghurts were significantly higher than for bovine yoghurts. Other studies have also reported comparable discrepancies in pH values between bovine and ovine yoghurt [34]. The observed differences in pH values between bovine and ovine yoghurts can be attributed to differences in their buffering capacities [35]. The dissimilarities in the pH values observed in bovine and ovine yoghurts can be attributed to variations in microbial growth, peptidase activity, and acidity evolution. Furthermore, these results suggest that fermentation was more effective in plain yoghurt samples as lactose content decreased in samples with increasing HPCF addition. This less-intensive acidification in fortified yoghurts can be also explained by the buffering capability of the hemp seed matrices [36]. A significant increase in pH values for bovine and ovine yoghurt samples was also observed with increasing HPCF addition (F (5, 96) = 112.1, *p* < 0.001) and a significant decrease in pH during storage (F (3, 96) = 36.0, *p* < 0.001). In addition, there was a statistically significant interaction between the effects of yoghurt type and HPCF addition (F (5, 96) = 5.4, *p* < 0.001), as well between yoghurt type and storage period (F (3, 96) = 4.9, *p* = 0.005).

A decrease in pH value when adding different plant components to yoghurt has been observed in the literature. In this regard, in yoghurt enriched with coriander and cumin seeds, the pH of the control yoghurt immediately upon its production was 4.56. Subsequently, the addition of coriander and cumin seeds in amounts of 5 and 20% reduced the pH value of the fortified yoghurt to 4.47 and 4.48, respectively [37]. Further, Sahingil and Hayaloglu (2022) [38] postulated that the addition of rosehip pulp (up to 20%) to yoghurt stored at 4 °C for 15 days reduced its pH value. This pH decrease trend might be explained by the stimulating effect of the yoghurt culture used and its metabolic activities. The ratio of organic acids, particularly lactic and acetic acids formed during normal metabolism of the cultures upon milk fermentation, ranged from 75 to 95% of the total metabolites depending on fermentation type [39]. The above-cited and the present results are important when preparing plant-enriched yoghurts, since the time of incubation taken to achieve the coagulation point changes dramatically compared to the plain yoghurt (without HPCF), thus leading to a possible modification in the product quality.

### 3.2. Change of Titratable Acidity

**Figure 3:**
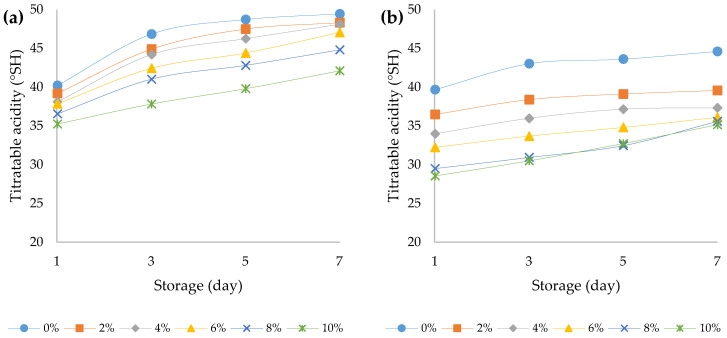
Titratable acidity over the course of storage days for (**a**) bovine and (**b**) ovine plain and fortified yoghurts with different percentage of HPCF.

The titratable acidity (TA) of the yoghurts is presented in Figure 3a,b. The TA of bovine and ovine plain yoghurts on the first day of production was 40.2 and 39.7 °*SH*, respectively. Significant differences (*p* < 0.05) in the TA of yoghurts with different amounts of HPCF was observed. The addition of HPCF to the yoghurts regardless of the milk origin caused a decrease in TA over a seven-day storage period. The outcomes of the three-way ANOVA indicated that all the principal factors and their interactions had statistically significant impacts (*p* < 0.05) (Table S2). The most prominent influence on TA was exerted by the type of yoghurt (F (1, 96) = 5229.0, *p* < 0.001) followed by the storage period (F (3, 96) = 837.1, *p* < 0.001) and the HPCF addition (F (5, 96) = 771.6, *p* < 0.001). When the addition of HPCF was increased, a relatively lower decrease in TA was recorded in bovine samples than in ovine samples (F (5, 96) = 69.2, *p* < 0.001), and during storage, the increase in TA was slower in ovine yoghurt samples (F (3, 96) = 89.9, *p* < 0.001). There was also a significant three-way interaction between main effects (F (15, 96) = 5.2, *p* < 0.001). The main reason for the observed changes in TA might be related to the yoghurt fermentation. This process continues during the storage of the yoghurt in cold conditions where an accumulation of lactic and other organic acids, including butyric, citric, acetic, formic acid, and acetaldehyde, might occur [40]. Szparaga et al. (2019) [41] reported that components derived from HPCF, including its biochemical compounds, phenols and flavonoids, can play an inhibitory role during yoghurt fermentation.

### 3.3. Colour Analysis

**Figure 4:**
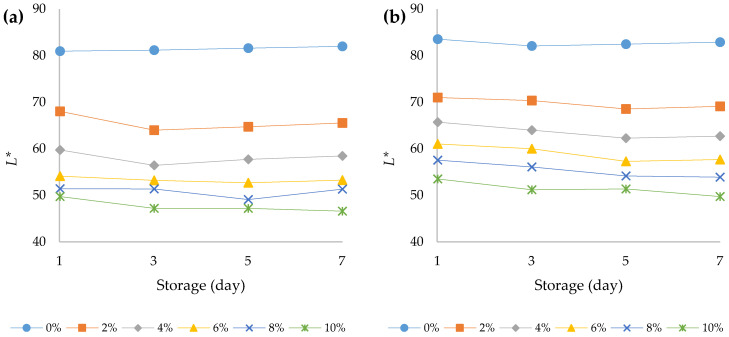
Variation in lightness values over the course of storage days for (**a**) bovine and (**b**) ovine plain and fortified yoghurts with different percentages of HPCF.

**Figure 5:**
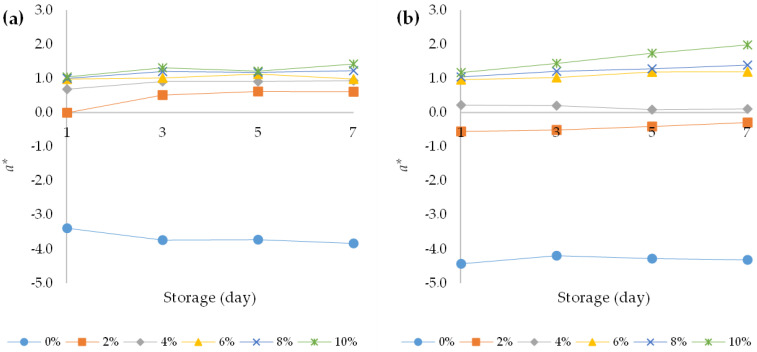
Changes in chromatic variable a* over the course of storage days for (**a**) bovine and (**b**) ovine plain and fortified yoghurts with different percentages of HPCF.

**Figure 6:**
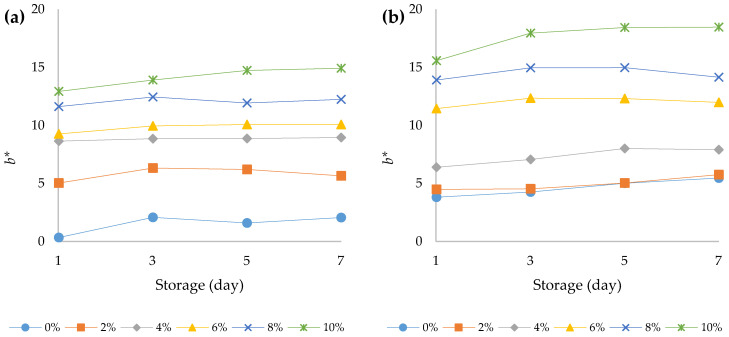
Changes in chromatic variable b* over the course of storage days for (**a**) bovine and (**b**) ovine plain and fortified yoghurts with different percentages of HPCF.

The choice of a food is mostly related to its colour and appearance, and these properties are functions of the food quality. Very often, colour is the first sensory characteristic that is perceived by the consumer and its change can lead to a change in consumer perception regarding its appearance, taste, and aroma [42]. According to the present study results, the addition of HPCF to the yoghurt samples caused significant decrease in the value of the L* parameter (*p* < 0.05) (Figure 4a,b). In addition, the prolonged storage period at 4 °C also led to a significant decrease (*p* < 0.05) in the values for this parameter. A similar trend was observed in the study conducted by Xu et al. (2022) [12]. Thus, both the addition of HPCF and the storage of yoghurt at 4 °C led to the creation of a darker colour of the fortified yoghurts. A three-way ANOVA revealed that HPCF addition had the greatest effect on yoghurt L* value (F (5, 96) = 2011.2, *p* < 0.001) followed by the effect of yoghurt type (F (1, 96) = 377.8, *p* < 0.001) and storage period (F (3, 96) = 21.6, *p* < 0.001). In addition, there was a statistically significant interaction between the effect of yoghurt type and HPCF addition (F (5, 96) = 9.1, *p* < 0.001), indicating that the decrease in L* value in the bovine yoghurt was more pronounced with increasing HPCF addition than in the ovine yoghurt (Table S3). Both the addition of HPCF and the storage of the yoghurt increased the a* value of the samples (Figure 5a,b). This increase was more pronounced in the bovine yoghurt samples, which was confirmed by the significant interaction between the yoghurt type and the amount of HPCF addition (F (5, 96) = 199.2, *p* < 0.001), and between the yoghurt type and the storage time (F (3, 96) = 3.7, *p* = 0.015). In addition, an interaction between HPCF addition and storage duration was also observed (F (15, 96) = 10.9, *p* < 0.001) (Table S4). Higher a* values of bovine yoghurt compared to ovine yoghurt were also observed in some other studies [43]. The value of the parameter b* in the bovine and ovine yoghurts increased with storage time and HPCF addition at 4 °C (Figure 6a,b). The results of the three-way ANOVA and post hoc Tukey’s HSD test showed that yoghurt type yielded an F ratio of F (1, 96) = 97.8 (*p* < 0.001), indicating that b* values for ovine yoghurts were significantly higher than for bovine yoghurts (Table S5). The greatest effect on the b* value was observed for HPCF addition (F (5, 96) = 720.5, *p* < 0.001). Storage time also had a significant effect, as did the interaction between yoghurt type and the amount of HPCF added (F (5, 96) = 35.2, *p* < 0.001). The b* values, as shown in Figure 6a,b, were positive for all samples regardless of the percentage of HPCF added, indicating a yellowish hue in the fortified yoghurts due to HPCF supplementation.

This result is consistent with previous studies. The colour of fermented beverages is often determined by the presence of pigments in the raw materials used, as reported by Olukomaiya et al. (2020) [44]. In addition, changes in storage time and pH can also impact the final colour of fermented foods, as demonstrated by Łopusiewicz et al. (2019) [45]. These authors found that the colour of fermented samples changed over time, and the variations in storage time and pH were identified as contributing factors. Similarly, Santos et al. (2019) [46] found that the optimization of soymilk fermentation with kefir and inulin resulted in a significant increase in the lightness of the fermented soymilk beverage. These findings demonstrate the importance of considering factors such as storage time and pH in the production of fermented beverages to ensure a consistent and desirable final colour.

The study conducted by Pojić et al. [47] showed that the addition of HPCF significantly decreased the lightness, redness, and yellowness of the bread crust. As the proportion of HPCF in the mixture increased, the lightness and yellowness of the bread crumb became less pronounced when compared with the control bread. In the study of Feng et al. (2022) [10] the lightness of potato chips was observed to significantly decrease with the addition of higher levels of HCP, resulting in a darker colour. This colour change is attributed to the darker shade of HCP. Similar results have been observed in other studies, where the lightness decreased upon adding HCP to meatballs [5] and to an extruded rice flour mixture [48]. These findings suggest that the incorporation of plant-based materials, such as HCP, can have a substantial impact on the colour of food products.

### 3.4. Total Phenolic Content and Antioxidant Activity

**Figure 7:**
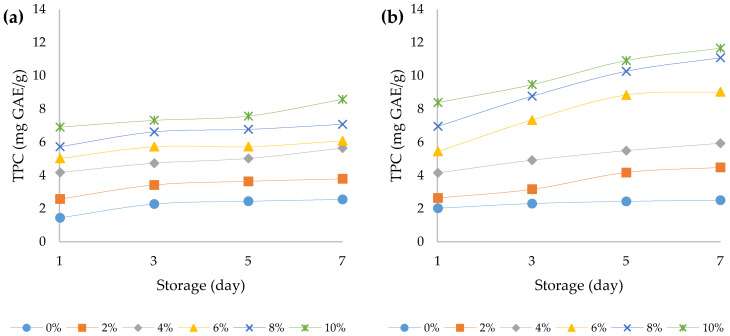
Total phenolic content (expressed as mg GAE/g) over the course of storage days for (**a**) bovine and (**b**) ovine plain and fortified yoghurts with different percentages of HPCF.

**Figure 8:**
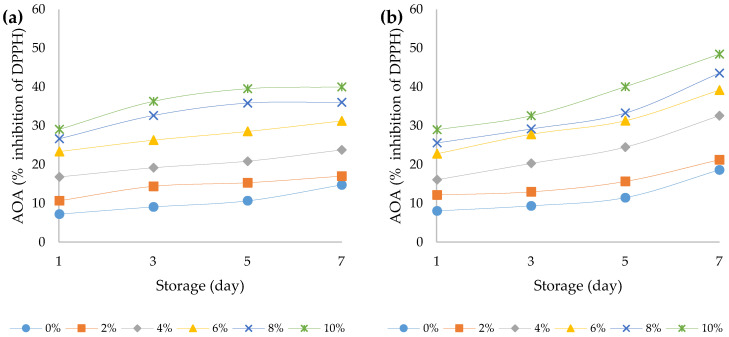
Antioxidant activity (expressed as percentage of Inhibition of DPPH) over the course of storage days for (**a**) bovine and (**b**) ovine plain and fortified yoghurts with different percentage of HPCF.

It has been found in the literature that *Cannabis Sativa* L. contains a large number of secondary metabolites, including terpenoids, cannabinoids, polyphenols (phenylamides, lignanamides and prenated flavonoids), fatty acids, amino acids, enzymes, sterols, pigments, and vitamins [49]. Nevertheless, a large part of these bioactive compounds remain in the hemp press cake even after the separation of the oil [17]. Recently, a strong correlation was established between the intake of polyphenols and a reduced incidence of chronic diseases, such as neurodegenerative diseases, cancer, and cardiovascular disorders [50]. The results of the determination of total polyphenol content and antioxidant activity of bovine and ovine yoghurts with different amounts of HPCF during seven days of storage are shown in Figure 7a,b and Figure 8a,b. The addition of HPCF significantly (*p* < 0.05) increased the total phenolic content compared to its content in the plain yoghurts (1.45 mg GAE/g in bovine plain yoghurt, 6.91 mg GAE/g in bovine yoghurt with 10% HPCF; 2.03 mg GAE/g in ovine plain yoghurt and 8.39 mg GAE/g in ovine yoghurt with 10% HPCF). The results of the three-way ANOVA showed that all main effects and their interactions had a statistically significant effect on the total phenolic content and antioxidant activity (*p* < 0.05) (Tables S6 and S7). The addition of HPCF had the strongest effect, both on the total phenolic content (F (5, 96) = 1307.5, *p* < 0.001) and antioxidant activity (F (5, 96) = 929.8, *p* < 0.001). Similar results were obtained in the study by Besir et al. (2022), where Aryan samples consisting of the mixture of bovine and hemp seed milk had higher phenolic content than the control Aryan sample. They explain this by the effect of proteolysis of hemp seed proteins and the release of amino acids with phenolic side chains, which contribute to the increase in the total phenolic content [51]. In our study the interaction between the effects of yoghurt type and HPCF addition was also significant (F (5, 96) = 35.2, *p* < 0.001). The significant increase in the total phenolic content was also observed during the prolonged storage of the yoghurts (F (3, 96) = 13.6, *p* < 0.001). Similarly, Shori (2020) [37] established that prolonged storage time at refrigerator temperature tremendously impacted the total phenolic content in yoghurt (*p* < 0.05). Additionally, Amirdivani and Baba (2011) [52] postulated that the phenolic content of plain yoghurt can be explained by the naturally occurring phenolic amino acid tyrosine, released during the proteolysis of milk proteins.

The biotransformation that takes place during milk fermentation can lead to a number of reactions that can possibly activate bioactive compounds that can act as natural antioxidants [17]. Antioxidants are substances that prevent the degradation or oxidation of compounds by donating an electron to free radicals and converting the latter into harmless compounds [53]. Total antioxidant activity is one of the most important parameters for determining the quality of medicinal plants (such as *Cannabis Sativa* L.) and for this determination, different in vitro antioxidant testing methods have been used [38]. The antioxidant activity expressed as a percentage of inhibition of DPPH radicals was also observed during the storage of the non-fortified and fortified yoghurt samples. From Figure 7a,b, it can be noticed that by increasing the amount of HPCF in the samples, the antioxidant activity also increased (from 7.23 and 8.03 in yoghurt samples with 0% HPC to 27.07 and 17.89 in yoghurts with 10% HPCF from bovine and ovine milks, respectively, determined on the first day after yoghurt production). One plausible explanation might be related to the metabolic activity of microorganisms, since it is known that fermentation also causes the structural breakdown of the microorganisms’ cell wall, which might lead to the synthesis of various bioactive compounds characterised with a high antioxidant potential [54]. The promotor antioxidant substances are actually found to remain in the hemp press cake after the seeds are pressed and following hemp oil production [17]. An increase in the antioxidant activity by adding rosehip pulp to a yoghurt (in amounts of 5, 10, 15, and 20%) was also confirmed by Sahingil and Hayaloglu (2022) [38]. The authors determined that the yoghurts enriched with rosehip pulp at 4 °C led to a tremendous increase in the antioxidant activity in the samples [38].

### 3.5. Microbiological Analysis

**Figure 9:**
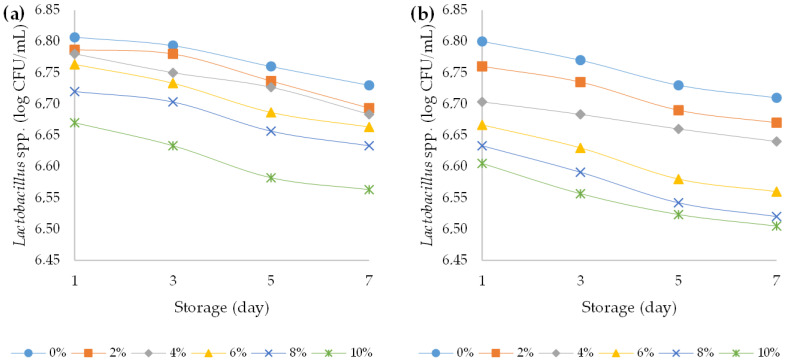
Variation in bacterial count of *Lactobacillus* spp. strains over the course of storage days for (**a**) bovine and (**b**) ovine plain and fortified yoghurts with different percentages of HPCF.

**Figure 10:**
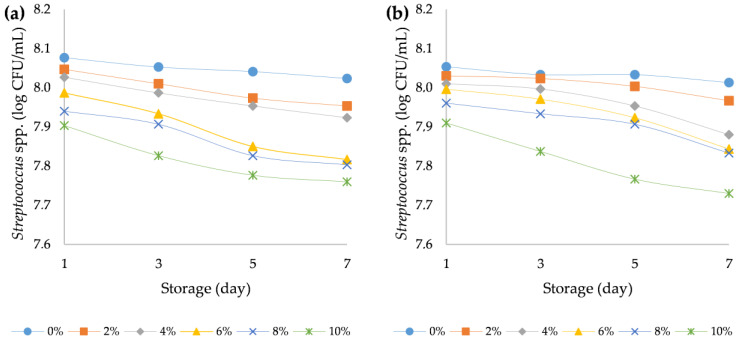
Variation in bacterial count of *Streptococcus* spp. strains over the course of storage days for (**a**) bovine and (**b**) ovine plain and fortified yoghurts with different percentages of HPCF.

**Figure 11:**
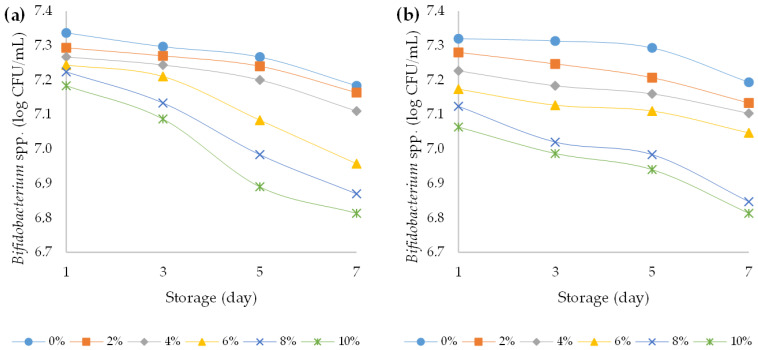
Variation in bacterial count of *Bifidobacterium* spp. strains over the course of storage days for (**a**) bovine and (**b**) ovine plain and fortified yoghurts with different percentages of HPCF.

Yoghurt is prepared by milk fermentation with three types of bacteria, namely *Lactobacillus acidophilus* (LA-5)^®^, *Bifidobacterium animalis* subsp. *lactis* (BB-12)^®^, and *Streptococcus thermophilus*; all other microorganisms present should be considered contaminants. The primary spoilage organisms in cultured milk (yoghurt, sour cream, and buttermilk) are yeasts and moulds, because the high acidity of these products inhibits the growth of many bacteria [55,56]. In the present study, all yoghurts during the storage time showed that the number of desired bacteria remained above 6 log CFU/mL. Namely, it is necessary to maintain the probiotic bacteria numbers above 6 log CFU/mL to give the beneficial probiotic effect (Figure 9, Figure 10 and Figure 11). Particularly, the viability of strains after the storage period was sufficient to yield numbers of beneficial organisms that were higher than the accepted threshold for a probiotic effect [57]. At the beginning of the storage period, *Streptococcus thermophilus* (about 8.05 log CFU/mL) were the most abundant in all samples, followed by *Bifidobacterium animalis* subsp. *lactis* (7.3 log CFU/mL) and *Lactobacillus acidophilus* (about 6.8 log CFU/mL). A three-way ANOVA showed that the addition of HPCF had a statistically significant (*p* < 0.5) effect on reducing the bacterial counts of Lactobacillus acidophilus (F (5, 96) = 26.5, *p* < 0.001), Streptococcus thermophilus (F (5, 96) = 64.4, *p* < 0.001), and *Bifidobacterium animalis* subsp. *lactis* (F (5, 96) = 105.1, *p* < 0.001) (Tables S8–S10).

This is most likely due to the presence of antimicrobial compounds found in the hemp [58]. The total number of natural compounds identified in hemp was stablished to be greater than 500, possessing the potential to exhibit antibacterial activity [59,60]. The Supplementary Material (Table S1) provides additional information on the viability of bacterial starter cultures, as well as the presence of pathogenic and spoilage microorganisms in yogurt samples collected during the storage period.

During storage of yoghurt, the viability of starter culture is an important factor affecting the safety of the final product. High acidity in products can protect consumers from food-borne pathogens. Microorganisms can produce a wide spectrum of organic acids, in particular, lactic-acid bacteria that are capable of producing lactic acid [61]. The therapeutic minimum of live microorganisms in the form of viable lactic acid bacteria in fermented foods should be 6 log CFU/mL [61]. In our case, the fermentation process during the seven-day storage decreased the content of viable lactic acid bacteria in all yoghurt samples. This was confirmed by the results of three-way ANOVA. Storage time had a statistically significant (*p* < 0.5) effect on reducing the bacterial counts of *Lactobacillus acidophilus* (F (3, 96) = 14.7, *p* < 0.001), *Streptococcus thermophilus* (F (3, 96) = 36.4, *p* < 0.001), and *Bifidobacterium animalis* subsp. *lactis* (F (3, 96) = 89.8, *p* < 0.001). A similar decrease in the lactic acid bacteria content after milk fermentation during storage was described in fermented hemp paste by Bartkiene et al. (2020) [62]. Compared to the ovine yogurt samples, the bovine yogurts had slightly but statistically significantly higher bacterial counts (*p* < 0.05) of *Lactobacillus acidophilus* (F (5, 96) = 36.4, *p* < 0.001) and *Bifidobacterium animalis* subsp. *lactis* (F (5, 96) = 8.3, *p* = 0.005) (Tables S8 and S10). Similar results were obtained in the study by Vianna et al. (2019), where bovine yoghurt samples had higher bacterial counts compared to ovine samples during 28 days of storage [63]. Total aerobic mesophilic bacteria are hygiene indicator microorganisms. These bacteria can provide information about the possible shelf life of food and contamination levels in the production stages [64]. Presence of several potential pathogenic bacteria (*Staphylococcus aureus*, *Escherichia coli*, and other *Enterobacteriaceae*) might be the proof of ineffective heat treatment or post-contamination of yoghurt [64]. In the present study, the presence of total viable bacteria, *Enterobacteria*, *Escherichia coli*, and *Staphylococcus aureus* was not detected in all analysed samples (plain and fortified bovine and ovine yoghurts) during the storage period, declaring the efficiency of the heat treatment and the absence of post-contamination of the yoghurt. The lactic acid bacteria in yoghurt produced lactic acid which prohibited the growth of contaminating microbiota [65].

**Figure 12:**
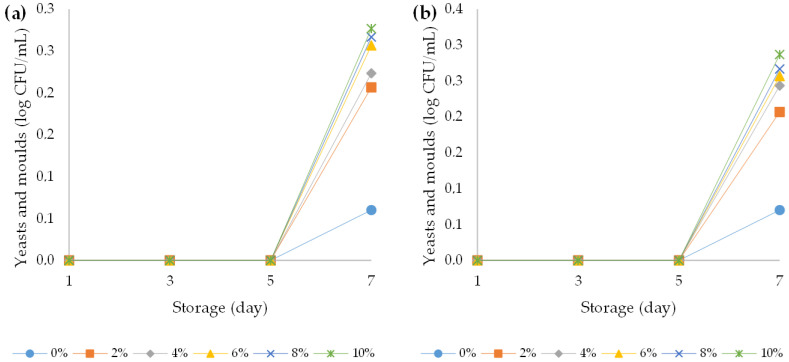
Variation in yeast and mould count over the course of storage days for (**a**) bovine and (**b**) ovine plain and fortified yoghurts with different percentages of HPCF.

Mould contamination is almost exclusively caused by the *Mucoraceae* family (*Saccharomyces cerevisiae* and *Kluyveromyces fragilis*), which has very strong proteolytic and lipolytic activity that leads to an intense odour in the yoghurts [66,67]. Contamination with yeasts is one of the main limiting factors for the stability and the commercial value of yoghurts. Spoilage becomes evident when the yeast population reaches 5 to 6 log CFU/mL [56]. The amounts of yeast and mould in the plain yoghurt on the initial day of examination were undetectable. The amounts of yeast and mould in all fortified yoghurts were higher than 0.2 log CFU/mL after day five of storage. In contrast, the total amounts of yeast and mould in the plain yoghurts in the whole storage time was very low (less than 0.1 log CFU/mL after day five of storage) (Figure 12). The results of the three-way ANOVA showed that storage duration had the greatest effect on the amounts of yeast and mould (F (3, 96) = 14367.4, *p* < 0.001) followed by the influence of HPCF addition (F (5, 96) = 318.4, *p* < 0.001) (Table S11). During this experiment, no differences were observed between yoghurt produced from bovine and ovine milks (*p* > 0.05).

### 3.6. Sensory Evaluation

**Figure 13:**
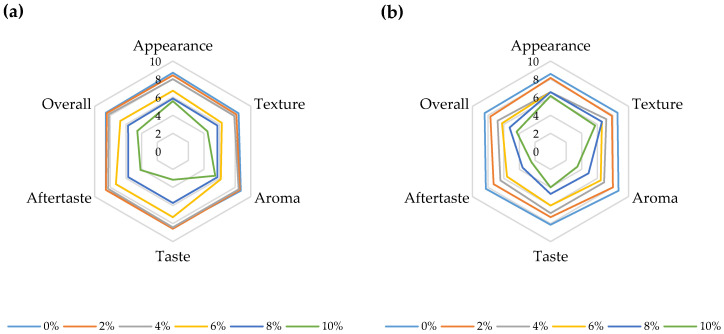
Sensory scores for (**a**) bovine and (**b**) ovine plain and fortified yoghurts with different percentages of HPCF.

Figure 13 presents the results of the sensory evaluation of the yoghurt samples. Generally, the control trials (yoghurts without any addition of HPCF) were the best evaluated with greater grades. It can be postulated that this is due to the fact that fermented products enriched with biologically active substances are not yet very accessible nor available on the market and the population is not in the habit of consuming those products [13,68]. The results of the three-way ANOVA showed that HPCF addition had the most significant effect on all sensory attributes (F (5, 72) = 196.2, *p* < 0.001 for overall score) (Table S12). The addition of hemp had the greatest impact on taste and aftertaste, where ratings decreased from 8.6 for the bovine yoghurt sample with 2% added HPMC to 3.1 for taste and 4.1 for aftertaste in the bovine sample with 10% HPCF and from 7.3 for the ovine sample with 2% HPCF to 4.0 for taste and 2.4 for aftertaste in the ovine sample with 10% HPCF. This decreased score for taste can be assigned to a lower acidity of fortified samples, as well as to the bitter taste of HPCF. Tukey’s HSD test revealed that there were no statistically significant differences between the control yoghurts and the samples with 4% added HPCF in terms of overall sensory score. The appearance score decreased with the increasing HPCF addition mainly due to a colour change as stated by the panellists.

Dabija et al. (2018) [69] found that the addition of hemp seeds, which contain 2.79% protein, to bovine yoghurt resulted in an overall score of 18.6 out of 20, suggesting that the product may be well received by consumers. The findings highlight the potential benefits of incorporating hemp seeds into yoghurt or other dairy products. Adding plant-based proteins, such as hemp seeds or pumpkin seed flour, can enhance the sensory qualities and nutritional value of bovine yoghurt. Furthermore, the addition of these plant proteins does not hinder the fermentation process or reduce the protein concentration of the final product. In conclusion, incorporating vegetable-based protein sources into dairy products has the potential to play a crucial role in the development of new and innovative dairy products.

Recently, our research group observed the sensory analysis of yoghurts fortified with HPCF and the addition of honey as a sweetener. The sensory evaluation of these products showed that the yoghurt containing 4% HPCF possessed the greatest sensory characteristics compared with the yoghurts carrying either higher, lower, or no presence of HPCF [20].

## 4. Conclusions

The results showed that the addition of HPCF significantly impacted the characteristics of the samples. The pH value of the yoghurts increased, and titratable acidity decreased as the amount of HPCF was increased. A small amount of HPCF caused a significant difference (*p* < 0.05) in colour, resulting in a darker, reddish, and yellowish hue. The addition of HPCF resulted in an increase in the total polyphenols and antioxidant activity in the samples. In this regard, it was revealed that the content of total polyphenols and antioxidant activity increased with continued storage of the samples at 4 °C. There were no statistically significant differences between the control yoghurts and the samples with 4% added HPCF in terms of overall sensory score. The yoghurt fortified with hemp press cakes was successfully manufactured with viable starter counts during the seven-day study period. However, the survival of starter cultures in yoghurt treatments was higher in samples with 0% hemp press cakes compared with the fortified yoghurts, as the growth of the microorganisms might have been inhibited by the antimicrobials from hemp press cakes.

Further research is required to comprehend the impact of HPCF on the quality and health benefits of fortified milk yoghurts. Future studies should encompass structural, functional, and digestion analyses both in vitro and in vivo to establish a clear connection between fortified milk yoghurts, HPCF, and human health. There is limited literature in this field; however, existing studies indicate the potential health benefits of HPCF, prompting us to make this recommendation. Additionally, more yoghurt samples need to be analysed in future studies, which should also include rheology, structure, and texture evaluations to gain a thorough understanding of the food-functional properties of fortified yoghurts and the true effect of HPCF on sustainable food by-product management.

## Figures and Tables

**Table 1:** Mean value (±standard deviation) of chemical composition of raw bovine and ovine milks.

Parameters	Bovine Milk	Ovine Milk
Fats, %	3.57 ± 0.28	7.54 ± 0.02
Solids Not Fat (SNF), %	8.60 ± 0.21	9.63 ± 0.02
Density g/cm^3^	1.029 ± 0.02	1.036 ± 0.07
Lactose, %	4.53 ± 0.11	4.63 ± 0.01
Solids, %	0.60 ± 0.02	0.90 ± 0.00
Proteins, %	2.94 ± 0.08	4.45 ± 0.01

## Data Availability

Data is contained within the article.

## Supplementary Materials

The following supporting information can be downloaded at: https://www.mdpi.com/article/10.3390/foods12050958/s1, Table S1: The results of three-way ANOVA followed by the post hoc Tukey test for pH values of (a) bovine and (b) ovine plain and fortified yoghurts with different proportions of hemp press cake flour (HPCF) during the seven-day storage period; Table S2: The results of three-way ANOVA followed by the post hoc Tukey test for titratable acidity (°*SH*) of (a) bovine and (b) ovine plain and fortified yoghurts with different proportions of hemp press cake flour (HPCF) during the seven-day storage period; Table S3: The results of three-way ANOVA followed by the post hoc Tukey test for *L** value of (a) bovine and (b) ovine plain and fortified yoghurts with different proportions of hemp press cake flour (HPCF) during the seven-day storage period; Table S4: The results of three-way ANOVA followed by the post hoc Tukey test for *a** value of (a) bovine and (b) ovine plain and fortified yoghurts with different proportions of hemp press cake flour (HPCF) during the seven-day storage period; Table S5: The results of three-way ANOVA followed by the post hoc Tukey test for *b** value of (a) bovine and (b) ovine plain and fortified yoghurts with different proportions of hemp press cake flour (HPCF) during the seven-day storage period; Table S6: The results of three-way ANOVA followed by the post hoc Tukey test for total phenolic content (TCP) in (a) bovine and (b) ovine plain and fortified yoghurts with different proportions of hemp press cake flour (HPCF) during the seven-day storage period; Table S7: The results of three-way ANOVA followed by the post hoc Tukey test for antioxidant activity (AOA) of (a) bovine and (b) ovine plain and fortified yoghurts with different proportions of hemp press cake flour (HPCF) during the seven-day storage period; Table S8: The results of three-way ANOVA followed by the post hoc Tukey test for bacterial count of *Lactobacillus* spp. strains in (a) bovine and (b) ovine plain and fortified yoghurts with different proportions of hemp press cake flour (HPCF) during the seven-day storage period; Table S9: The results of three-way ANOVA followed by the post hoc Tukey test for bacterial count of *Streptococcus* spp. strains in (a) bovine and (b) ovine plain and fortified yoghurts with different proportions of hemp press cake flour (HPCF) during the seven-day storage period; Table S10: The results of three-way ANOVA followed by the post hoc Tukey test for bacterial count of *Bifidobacterium* spp. strains in (a) bovine and (b) ovine plain and fortified yoghurts with different proportions of hemp press cake flour (HPCF) during the seven-day storage period; Table S11: The results of three-way ANOVA followed by the post hoc Tukey test for count of yeasts and moulds in (a) bovine and (b) ovine plain and fortified yoghurts with different proportions of hemp press cake flour (HPCF) during the seven-day storage period; Table S12: The results of three-way ANOVA followed by the post hoc Tukey test of sensory scores in (a) bovine and (b) ovine plain and fortified yoghurts with different proportions of hemp press cake flour (HPCF).
