# Supplementary material for: The Influence of the Addition of Hemp Press Cake Flour on the Properties of Bovine and Ovine Yoghurts
Source: Foods. 2023 Feb 24;12(5):958. doi: 10.3390/foods12050958 (PMC10001388; doi:10.3390/foods12050958)
Supplement: Supplementary file 1 [file foods-12-00958-s001.zip › foods-2190701-supplementary.pdf]

**Table S1:** The results of Three-way ANOVA followed by the post-hoc Tukey test for pH values of a) bovine and b) ovine plain and fortified yoghurts with different proportions of hemp press cake flour (HPCF) during the 7-day storage period.

| Source | DF | SS | MS | *F* | *p*[1] | Storage (day) | pH[2] | HPCF (%) | pH[2] |
|---|---|---|---|---|---|---|---|---|---|
| Type (1) | 1 | 0.496 | 0.496 | 388.204 | **< 0,0001** | 1 | 4.63[a] | 0 | 4.45[f] |
| HPCF (2) | 5 | 0.716 | 0.143 | 112.117 | **< 0,0001** | 3 | 4.58[b] | 2 | 4.49[e] |
| Storage (3) | 3 | 0.138 | 0.046 | 36.043 | **< 0,0001** | 5 | 4.55[bc] | 4 | 4.54[d] |
| (1) x (2) | 5 | 0.035 | 0.007 | 5.421 | **0.000** | 7 | 4.53[c] | 6 | 4.61[c] |
| (1) x (3) | 3 | 0.019 | 0.006 | 4.867 | **0.005** | **Type** | | 8 | 4.65[b] |
| (2) x (3) | 15 | 0.003 | 0.000 | 0.164 | 1.000 | Bovine | 4.50[b] | 10 | 4.69[a] |
| (1) x (2) x (3) | 15 | 0.016 | 0.001 | 0.842 | 0.629 | Ovine | 4.64[a] | | |

[1]Bolded *p*-values are statistically significant ($p < 0.05$)

[2]Different letters indicate statistically significant differences ($p < 0.05$) separately for storage time, type of the yogurt and level of the HPCF addition

**Table S2:** The results of Three-way ANOVA followed by the post-hoc Tukey test for Titratable Acidity (°SH) of a) bovine and b) ovine plain and fortified yoghurts with different proportions of hemp press cake flour (HPCF) during the 7-day storage period.

| Source | DF | SS | MS | *F* | *p*[1] | Storage (day) | TA (°SH)[2] | HPCF (%) | TA (°SH)[2] |
|---|---|---|---|---|---|---|---|---|---|
| Type (1) | 1 | 1241.1 | 1241.1 | 5229.3 | **< 0,0001** | 1 | 35.6[d] | 0 | 44.5[a] |
| HPCF (2) | 5 | 915.7 | 183.1 | 771.6 | **< 0,0001** | 3 | 39.1[c] | 2 | 41.7[b] |
| Storage (3) | 3 | 596.0 | 198.7 | 837.1 | **< 0,0001** | 5 | 40.8[b] | 4 | 40.1[c] |
| (1) x (2) | 5 | 82.1 | 16.4 | 69.2 | **< 0,0001** | 7 | 42.3[a] | 6 | 38.6[d] |
| (1) x (3) | 3 | 64.0 | 21.3 | 89.9 | **< 0,0001** | **Type** | | 8 | 36.7[e] |
| (2) x (3) | 15 | 17.0 | 1.1 | 4.8 | **< 0,0001** | Bovine | 43.1[a] | 10 | 35.2[f] |
| (1) x (2) x (3) | 15 | 18.7 | 1.2 | 5.2 | **< 0,0001** | Ovine | 35.9[b] | | |

[1]Bolded *p*-values are statistically significant ($p < 0.05$)

[2]Different letters indicate statistically significant differences ($p < 0.05$) separately for storage time, type of the yogurt and level of the HPCF addition

**Table S3:** The results of Three-way ANOVA followed by the post-hoc Tukey test for *L** value of a) bovine and b) ovine plain and fortified yoghurts with different proportions of hemp press cake flour (HPCF) during the 7-day storage period.

| Source | DF | SS | MS | *F* | *p*[1] | Storage (day) | *L**[2] | HPCF (%) | *L**[2] |
|---|---|---|---|---|---|---|---|---|---|
| Type (1) | 1 | 634.9 | 634.9 | 377.8 | **< 0,0001** | 1 | 63.0[a] | 0 | 82.1[a] |
| HPCF (2) | 5 | 16896.0 | 3379.2 | 2011.2 | **< 0,0001** | 3 | 61.4[b] | 2 | 67.7[b] |
| Storage (3) | 3 | 108.8 | 36.3 | 21.6 | **< 0,0001** | 5 | 61.1[b] | 4 | 60.9[c] |
| (1) x (2) | 5 | 76.6 | 15.3 | 9.1 | **< 0,0001** | 7 | 60.8[b] | 6 | 56.1[d] |
| (1) x (3) | 3 | 20.3 | 6.8 | 4.0 | **0.009** | **Type** | | 8 | 53.1[e] |
| (2) x (3) | 15 | 41.6 | 2.8 | 1.6 | 0.075 | Bovine | 59.5[b] | 10 | 49.6[f] |
| (1) x (2) x (3) | 15 | 22.5 | 1.5 | 0.9 | 0.574 | Ovine | 63.7[a] | | |

[1]Bolded *p*-values are statistically significant ($p < 0.05$)

[2]Different letters indicate statistically significant differences ($p < 0.05$) separately for storage time, type of the yogurt and level of the HPCF addition

**Table S4:** The results of Three-way ANOVA followed by the post-hoc Tukey test for *a** value of a) bovine and b) ovine plain and fortified yoghurts with different proportions of hemp press cake flour (HPCF) during the 7-day storage period.

| **Source** | **DF** | **SS** | **MS** | ***F*** | ***p*[1]** | **Storage (day)** | ***a**[2]** | **HPCF (%)** | ***a**[2]** |
|---|---|---|---|---|---|---|---|---|---|
| Type (1) | 1 | 3.0 | 3.0 | 385.9 | **< 0,0001** | 1 | -0.11$^{c}$ | 0 | -3.99$^{f}$ |
| HPCF (2) | 5 | 496.3 | 99.3 | 12811.0 | **< 0,0001** | 3 | 0.03$^{b}$ | 2 | 0.00$^{e}$ |
| Storage (3) | 3 | 1.0 | 0.3 | 44.0 | **< 0,0001** | 5 | 0.08$^{ab}$ | 4 | 0.51$^{d}$ |
| (1) x (2) | 5 | 7.7 | 1.5 | 199.2 | **< 0,0001** | 7 | 0.12$^{a}$ | 6 | 1.06$^{c}$ |
| (1) x (3) | 3 | 0.1 | 0.0 | 3.7 | **0.015** | **Type** | | 8 | 1.19$^{b}$ |
| (2) x (3) | 15 | 1.3 | 0.1 | 10.9 | **< 0,0001** | Bovine | 0.17$^{a}$ | 10 | 1.41$^{a}$ |
| (1) x (2) x (3) | 15 | 0.9 | 0.1 | 8.1 | **< 0,0001** | Ovine | -0.11$^{b}$ | | |

[1]Bolded *p*-values are statistically significant ($p < 0.05$)

[2]Different letters indicate statistically significant differences ($p < 0.05$) separately for storage time, type of the yogurt and level of the HPCF addition

**Table S5:** The results of Three-way ANOVA followed by the post-hoc Tukey test for *b** value of a) bovine and b) ovine plain and fortified yoghurts with different proportions of hemp press cake flour (HPCF) during the 7-day storage period.

| **Source** | **DF** | **SS** | **MS** | ***F*** | ***p*[1]** | **Storage (day)** | ***b**[2]** | **HPCF (%)** | ***b**[2]** |
|---|---|---|---|---|---|---|---|---|---|
| Type (1) | 1 | 76.5 | 76.5 | 97.8 | **< 0,0001** | 1 | 8.6b | 0 | 3.1f |
| HPCF (2) | 5 | 2818.4 | 563.7 | 720.5 | **< 0,0001** | 3 | 9.6a | 2 | 5.3e |
| Storage (3) | 3 | 31.8 | 10.6 | 13.6 | **< 0,0001** | 5 | 9.7a | 4 | 8.1d |
| (1) x (2) | 5 | 137.6 | 27.5 | 35.2 | **< 0,0001** | 7 | 9.8a | 6 | 10.9c |
| (1) x (3) | 3 | 0.8 | 0.3 | 0.3 | 0.811 | **Type** | | 8 | 13.3b |
| (2) x (3) | 15 | 13.0 | 0.9 | 1.1 | 0.361 | Bovine | 8.7b | 10 | 15.9a |
| (1) x (2) x (3) | 15 | 10.0 | 0.7 | 0.9 | 0.621 | Ovine | 10.2a | | |

[1]Bolded *p*-values are statistically significant ($p < 0.05$)

[2]Different letters indicate statistically significant differences ($p < 0.05$) separately for storage time, type of the yogurt and level of the HPCF addition

**Table S6:** The results of Three-way ANOVA followed by the post-hoc Tukey test for Total phenolic content (TCP) in a) bovine and b) ovine plain and fortified yoghurts with different proportions of hemp press cake flour (HPCF) during the 7-day storage period.

| **Source** | **DF** | **SS** | **MS** | ***F*** | ***p*[1]** | **Storage (day)** | **TPC (mg GAE/g) [2]** | **HPCF (%)** | **TPC (mg GAE/g) [2]** |
|---|---|---|---|---|---|---|---|---|---|
| Type (1) | 1 | 41.4 | 41.4 | 512.8 | **< 0,0001** | 1 | 4.6$^{d}$ | 0 | 2.2$^{f}$ |
| HPCF (2) | 5 | 528.4 | 105.7 | 1307.5 | **< 0,0001** | 3 | 5.5$^{c}$ | 2 | 3.5$^{e}$ |
| Storage (3) | 3 | 49.6 | 16.5 | 204.4 | **< 0,0001** | 5 | 6.1$^{b}$ | 4 | 5.0$^{d}$ |
| (1) x (2) | 5 | 30.4 | 6.1 | 75.2 | **< 0,0001** | 7 | 6.5$^{a}$ | 6 | 6.6$^{c}$ |
| (1) x (3) | 3 | 6.7 | 2.2 | 27.5 | **< 0,0001** | **Type** | | 8 | 7.9$^{b}$ |
| (2) x (3) | 15 | 6.9 | 0.5 | 5.7 | **< 0,0001** | Bovine | 5.0$^{b}$ | 10 | 8.9$^{a}$ |
| (1) x (2) x (3) | 15 | 6.0 | 0.4 | 5.0 | **< 0,0001** | Ovine | 6.3$^{a}$ | | |

[1]Bolded *p*-values are statistically significant ($p < 0.05$)

[2]Different letters indicate statistically significant differences ($p < 0.05$) separately for storage time, type of the yogurt and level of the HPCF addition

**Table S7:** The results of Three-way ANOVA followed by the post-hoc Tukey test for Antioxidant activity (AOA) of a) bovine and b) ovine plain and fortified yoghurts with different proportions of hemp press cake flour (HPCF) during the 7-day storage period.

| **Source** | **DF** | **SS** | **MS** | ***F*** | $p^1$ | **Storage (day)** | **AOA (% inhibition of DPPH)** [2] | **HPCF (%)** | **AOA (% inhibition of DPPH)** [2] |
|---|---|---|---|---|---|---|---|---|---|
| Type (1) | 1 | 75.8 | 75.8 | 42.9 | **< 0,0001** | 1 | 18.8d | 0 | 11.2f |
| HPCF (2) | 5 | 8212.4 | 1642.5 | 929.8 | **< 0,0001** | 3 | 22.5c | 2 | 14.9e |
| Storage (3) | 3 | 1781.2 | 593.7 | 336.1 | **< 0,0001** | 5 | 25.6b | 4 | 21.8d |
| (1) x (2) | 5 | 26.3 | 5.3 | 3.0 | **0.020** | 7 | 30.5a | 6 | 28.8c |
| (1) x (3) | 3 | 214.0 | 71.3 | 40.4 | **< 0,0001** | **Type** | | 8 | 32.9b |
| (2) x (3) | 15 | 132.5 | 8.8 | 5.0 | **< 0,0001** | Bovine | 23.5b | 10 | 36.6a |
| (1) x (2) x (3) | 15 | 55.0 | 3.7 | 2.1 | **0.029** | Ovine | 25.3a | | |

[1]Bolded *p*-values are statistically significant ($p < 0.05$)

[2]Different letters indicate statistically significant differences ($p < 0.05$) separately for storage time, type of the yogurt and level of the HPCF addition

**Table S8:** The results of Three-way ANOVA followed by the post-hoc Tukey test for bacterial count of *Lactobacillus* spp. strains in a) bovine and b) ovine plain and fortified yoghurts with different proportions of hemp press cake flour (HPCF) during the 7-day storage period.

| **Source** | **DF** | **SS** | **MS** | ***F*** | $p^1$ | **Storage (day)** | ***Lactobacillus* spp. (log CFU/mL)**[2] | **HPCF (%)** | ***Lactobacillus* spp. (log CFU/mL)**[2] |
|---|---|---|---|---|---|---|---|---|---|
| Type (1) | 1 | 0.154 | 0.154 | 36.401 | **< 0,0001** | 1 | 6.72$^{a}$ | 0 | 6.76$^{a}$ |
| HPCF (2) | 5 | 0.562 | 0.112 | 26.530 | **< 0,0001** | 3 | 6.70$^{a}$ | 2 | 6.73$^{ab}$ |
| Storage (3) | 3 | 0.187 | 0.062 | 14.749 | **< 0,0001** | 5 | 6.66$^{b}$ | 4 | 6.70$^{bc}$ |
| (1) x (2) | 5 | 0.036 | 0.007 | 1.715 | 0.138 | 7 | 6.63$^{b}$ | 6 | 6.66$^{cd}$ |
| (1) x (3) | 3 | 0.001 | 0.000 | 0.085 | 0.968 | **Type** | | 8 | 6.62$^{de}$ |
| (2) x (3) | 15 | 0.004 | 0.000 | 0.070 | 1.000 | Bovine | 6.71$^{a}$ | 10 | 6.58$^{e}$ |
| (1) x (2) x (3) | 15 | 0.002 | 0.000 | 0.034 | 1.000 | Ovine | 6.64$^{b}$ | | |

[1]Bolded *p*-values are statistically significant ($p < 0.05$)

[2]Different letters indicate statistically significant differences ($p < 0.05$) separately for storage time, type of the yogurt and level of the HPCF addition

**Table S9:** The results of Three-way ANOVA followed by the post-hoc Tukey test for bacterial count of *Streptococcus* spp. strains in a) bovine and b) ovine plain and fortified yoghurts with different proportions of hemp press cake flour (HPCF) during the 7-day storage period.

| **Source** | **DF** | **SS** | **MS** | ***F*** | $p^1$ | **Storage (day)** | ***Streptococcus* spp. (log CFU/mL)**[2] | **HPCF (%)** | ***Streptococcus* spp. (log CFU/mL)**[2] |
|---|---|---|---|---|---|---|---|---|---|
| Type (1) | 1 | 0.003 | 0.003 | 1.108 | 0.295 | 1 | 7.99$^{a}$ | 0 | 8.04$^{a}$ |
| HPCF (2) | 5 | 0.809 | 0.162 | 64.364 | **< 0,0001** | 3 | 7.96$^{b}$ | 2 | 8.00$^{ab}$ |
| Storage (3) | 3 | 0.274 | 0.091 | 36.394 | **< 0,0001** | 5 | 7.92$^{c}$ | 4 | 7.97$^{b}$ |
| (1) x (2) | 5 | 0.018 | 0.004 | 1.405 | 0.229 | 7 | 7.88$^{d}$ | 6 | 7.92$^{c}$ |
| (1) x (3) | 3 | 0.006 | 0.002 | 0.766 | 0.516 | **Type** | | 8 | 7.89$^{c}$ |
| (2) x (3) | 15 | 0.045 | 0.003 | 1.189 | 0.294 | Bovine | 7.93$^{a}$ | 10 | 7.81$^{d}$ |
| (1) x (2) x (3) | 15 | 0.007 | 0.000 | 0.181 | 1.000 | Ovine | 7.94$^{a}$ | | |

[1]Bolded *p*-values are statistically significant ($p < 0.05$)

[2]Different letters indicate statistically significant differences ($p < 0.05$) separately for storage time, type of the yogurt and level of the HPCF addition

**Table S10:** The results of Three-way ANOVA followed by the post-hoc Tukey test for bacterial count of *Bifidobacterium* spp. strains in a) bovine and b) ovine plain and fortified yoghurts with different proportions of hemp press cake flour (HPCF) during the 7-day storage period.

| **Source** | **DF** | **SS** | **MS** | ***F*** | ***p*[1]** | **Storage (day)** | ***Bifidobacterium* spp. (log CFU/mL)[2]** | **HPCF (%)** | ***Bifidobacterium* spp. (log CFU/mL)[2]** |
|---|---|---|---|---|---|---|---|---|---|
| Type (1) | 1 | 0.027 | 0.027 | 8.281 | **0.005** | 1 | $7.23^{a}$ | 0 | $7.28^{a}$ |
| HPCF (2) | 5 | 1.695 | 0.339 | 105.120 | **< 0,0001** | 3 | $7.18^{b}$ | 2 | $7.23^{ab}$ |
| Storage (3) | 3 | 0.869 | 0.290 | 89.862 | **< 0,0001** | 5 | $7.11^{c}$ | 4 | $7.19^{b}$ |
| (1) x (2) | 5 | 0.018 | 0.004 | 1.118 | 0.356 | 7 | $7.02^{d}$ | 6 | $7.12^{c}$ |
| (1) x (3) | 3 | 0.039 | 0.013 | 4.071 | **0.009** | **Type** | | 8 | $7.02^{d}$ |
| (2) x (3) | 15 | 0.133 | 0.009 | 2.753 | **0.001** | Bovine | $7.15^{a}$ | 10 | $6.97^{e}$ |
| (1) x (2) x (3) | 15 | 0.039 | 0.003 | 0.803 | 0.672 | Ovine | $7.12^{b}$ | | |

[1]Bolded *p*-values are statistically significant ($p < 0.05$)

[2]Different letters indicate statistically significant differences ($p < 0.05$) separately for storage time, type of the yogurt and level of the HPCF addition

**Table S11:** The results of Three-way ANOVA followed by the post-hoc Tukey test for count of yeasts and moulds in a) bovine and b) ovine plain and fortified yoghurts with different proportions of hemp press cake flour (HPCF) during the 7-day storage period.

| **Source** | **DF** | **SS** | **MS** | ***F*** | ***p*[1]** | **Storage (day)** | **Yeasts and moulds (log CFU/mL)[2]** | **HPCF (%)** | **Yeasts and moulds (log CFU/mL)[2]** |
|---|---|---|---|---|---|---|---|---|---|
| Type (1) | 1 | 0.00 | 0.00 | 3.35 | 0.070 | 1 | $0.00^{b}$ | 0 | $0.016^{e}$ |
| HPCF (2) | 5 | 0.05 | 0.01 | 318.36 | **< 0,0001** | 3 | $0.00^{b}$ | 2 | $0.052^{d}$ |
| Storage (3) | 3 | 1.29 | 0.43 | 14367.35 | **< 0,0001** | 5 | $0.00^{b}$ | 4 | $0.058^{c}$ |
| (1) x (2) | 5 | 0.00 | 0.00 | 0.84 | 0.527 | 7 | $0.218^{a}$ | 6 | $0.064^{b}$ |
| (1) x (3) | 3 | 0.00 | 0.00 | 3.35 | **0.022** | **Type** | | 8 | $0.067^{ab}$ |
| (2) x (3) | 15 | 0.14 | 0.01 | 318.36 | **< 0,0001** | Bovine | $0.054^{a}$ | 10 | $0.070^{a}$ |
| (1) x (2) x (3) | 15 | 0.00 | 0.00 | 0.84 | 0.635 | Ovine | $0.055^{a}$ | | |

[1]Bolded *p*-values are statistically significant ($p < 0.05$)

[2]Different letters indicate statistically significant differences ($p < 0.05$) separately for storage time, type of the yogurt and level of the HPCF addition

**Table S12:** The results of Three-way ANOVA followed by the post-hoc Tukey test of sensory scores in a) bovine and b) ovine plain and fortified yoghurts with different proportions of hemp press cake flour (HPCF).

| Source | DF | SS | MS | *F* | *p*[1] | Type | Score[2] | HPCF (%) | Score[2] |
|---|---|---|---|---|---|---|---|---|---|
| ***Appearance*** | | | | | | Bovine | $7.2^{a}$ | 0 | $8.6^{a}$ |
| Type (1) | 1 | 0.3 | 0.3 | 0.4 | 0.522 | Ovine | $7.1^{a}$ | 2 | $8.3^{a}$ |
| HPCF (2) | 5 | 88.8 | 17.8 | 24.7 | **< 0,0001** | | | 4 | $7.3^{b}$ |
| (1) x (2) | 5 | 10.2 | 2.0 | 2.8 | **0.021** | | | 6 | $6.6^{bc}$ |
| | | | | | | | | 8 | $6.2^{c}$ |
| | | | | | | | | 10 | $5.9^{c}$ |
| ***Texture*** | | | | | | Bovine | $6.8^{a}$ | 0 | $8.3^{a}$ |
| Type (1) | 1 | 8.7 | 8.7 | 12.4 | **0.001** | Ovine | $6.2^{b}$ | 2 | $7.7^{ab}$ |
| HPCF (2) | 5 | 173.6 | 34.7 | 49.4 | **< 0,0001** | | | 4 | $7.4^{b}$ |
| (1) x (2) | 5 | 2.1 | 0.4 | 0.6 | 0.700 | | | 6 | $6.1^{c}$ |
| | | | | | | | | 8 | $5.2^{d}$ |
| | | | | | | | | 10 | $4.2^{e}$ |
| ***Aroma*** | | | | | | Bovine | $7.2^{a}$ | 0 | $8.6^{a}$ |
| Type (1) | 1 | 0.2 | 0.2 | 0.2 | 0.653 | Ovine | $7.1^{a}$ | 2 | $8.2^{a}$ |
| HPCF (2) | 5 | 110.5 | 22.1 | 23.7 | **< 0,0001** | | | 4 | $7.8^{a}$ |
| (1) x (2) | 5 | 11.0 | 2.2 | 2.3 | **0.049** | | | 6 | $6.4^{b}$ |
| | | | | | | | | 8 | $6.1^{b}$ |
| | | | | | | | | 10 | $5.6^{b}$ |
| ***Taste*** | | | | | | Bovine | $6.9^{a}$ | 0 | $8.6^{a}$ |
| Type (1) | 1 | 6.3 | 6.3 | 7.4 | **0.008** | Ovine | $6.4^{b}$ | 2 | $8.3^{a}$ |
| HPCF (2) | 5 | 288.1 | 57.6 | 67.5 | **< 0,0001** | | | 4 | $7.6^{ab}$ |
| (1) x (2) | 5 | 9.2 | 1.8 | 2.2 | 0.068 | | | 6 | $6.9^{b}$ |
| | | | | | | | | 8 | $5.3^{c}$ |
| | | | | | | | | 10 | $3.3^{d}$ |
| ***Aftertaste*** | | | | | | Bovine | $7.1^{a}$ | 0 | $8.4^{a}$ |
| Type (1) | 1 | 47.3 | 47.3 | 65.1 | **< 0,0001** | Ovine | $5.6^{b}$ | 2 | $7.9^{ab}$ |
| HPCF (2) | 5 | 281.9 | 56.4 | 77.6 | **< 0,0001** | | | 4 | $7.4^{bc}$ |
| (1) x (2) | 5 | 7.5 | 1.5 | 2.1 | 0.078 | | | 6 | $6.4^{c}$ |
| | | | | | | | | 8 | $4.6^{d}$ |
| | | | | | | | | 10 | $3.3^{e}$ |
| ***Overall*** | | | | | | Bovine | $7.0^{a}$ | 0 | $8.5^{a}$ |
| Type (1) | 1 | 7.1 | 7.1 | 40.1 | **< 0,0001** | Ovine | $6.5^{b}$ | 2 | $8.1^{a}$ |
| HPCF (2) | 5 | 173.5 | 34.7 | 196.2 | **< 0,0001** | | | 4 | $7.5^{ab}$ |
| (1) x (2) | 5 | 3.9 | 0.8 | 4.4 | **0.001** | | | 6 | $6.5^{b}$ |
| | | | | | | | | 8 | $5.5^{c}$ |
| | | | | | | | | 10 | $4.4^{d}$ |

[1]Bolded *p*-values are statistically significant ($p < 0.05$)

[2]Different letters indicate statistically significant differences ($p < 0.05$) separately for each sensory attribute
